# Antioxidant Effects of Korean Propolis in HaCaT Keratinocytes Exposed to Particulate Matter 10

**DOI:** 10.3390/antiox11040781

**Published:** 2022-04-14

**Authors:** In Ah Bae, Jae Won Ha, Joon Yong Choi, Yong Chool Boo

**Affiliations:** 1 Department of Biomedical Science, The Graduate School, Kyungpook National University, 680 Gukchaebosang-ro, Jung-gu, Daegu 41944, Korea; sksnadlv@naver.com (I.A.B.); jaewon1226@knu.ac.kr (J.W.H.); halo134679@naver.com (J.Y.C.); 2 BK21 Plus KNU Biomedical Convergence Program, Kyungpook National University, 680 Gukchaebosang-ro, Jung-gu, Daegu 41944, Korea; 3 Department of Molecular Medicine, School of Medicine, Kyungpook National University, 680 Gukchaebosang-ro, Jung-gu, Daegu 41944, Korea; 4 Cell and Matrix Research Institute, Kyungpook National University, 680 Gukchaebosang-ro, Jung-gu, Daegu 41944, Korea

**Keywords:** Korean propolis, particulate matter, oxidative stress, keratinocytes, ferulic acid, caffeic acid, *p-*coumaric acid

## Abstract

Air pollution causes oxidative stress that leads to inflammatory diseases and premature aging of the skin. The purpose of this study was to examine the antioxidant effect of Korean propolis on oxidative stress in human epidermal HaCaT keratinocytes exposed to particulate matter with a diameter of less than 10 μm (PM_10_). The total ethanol extract of propolis was solvent-fractionated with water and methylene chloride to divide into a hydrophilic fraction and a lipophilic fraction. The lipophilic fraction of propolis was slightly more cytotoxic, and the hydrophilic fraction was much less cytotoxic than the total extract. The hydrophilic fraction did not affect the viability of cells exposed to PM_10_, but the total propolis extract and the lipophilic fraction aggravated the toxicity of PM_10_. The total extract and hydrophilic fraction inhibited PM_10_-induced ROS production and lipid peroxidation in a concentration-dependent manner, whereas the lipophilic fraction did not show such effects. High-performance liquid chromatography with photodiode array detection (HPLC-DAD) analysis showed that the hydrophilic fraction contained phenylpropanoids, such as caffeic acid, *p-*coumaric acid, and ferulic acid, whereas the lipophilic faction contained caffeic acid phenethyl ester (CAPE). The former three compounds inhibited PM_10_-induced ROS production, lipid peroxidation, and/or glutathione oxidation, and ferulic acid was the most effective among them, but CAPE exhibited cytotoxicity and aggravated the toxicity of PM_10_. This study suggests that Korean propolis, when properly purified, has the potential to be used as a cosmetic material that helps to alleviate the skin toxicity of air pollutants.

## 1. Introduction

Industrial development and increased human activity are causing environmental pollution problems. In particular, air pollutants from natural and artificial sources cause fatal diseases, such as respiratory, cardiovascular, and brain-neurological diseases, and are an important cause of death for modern humans [1,2]. Air pollution has a detrimental effect on the health of the skin, the outermost organ of our body, and causes various inflammatory diseases, such as atopy, psoriasis, and acne, as well as premature skin aging [2,3]. Therefore, a dermatological or cosmetic defense strategy against air pollution should be devised to maintain skin health.

Air pollutants include gas components, such as ozone (O_3_), nitrogen dioxide (NO_2_), and sulfur dioxide (SO_2_), and suspended particulate matter of various compositions [4]. The suspended particulate matter with a size of less than 10 μm is called PM_10_, and it is a mixture of various organic compounds, such as aryl hydrocarbons, various heavy metals, such as cobalt, lead, and cadmium, and biological constituents [5,6]. PM_10_ can enter the body through various routes, such as the mouth, nose, eyes, and ears, and can also penetrate the skin through pores or the sites where the skin barrier is weak [7,8,9,10]. The components of PM_10_ generate reactive oxygen species (ROS) through chemical reactions or biological metabolism inside and outside cells [11,12,13,14], causing oxidative damage and inflammatory responses [15,16]. On the other hand, it is suggested that various types of antioxidants may help protect skin health by alleviating the oxidative stress and inflammatory response induced by PM_10_ [17].

Propolis is a natural product made by bees by mixing their discharges with the sap and pollen they collected from the plant. It is a green, yellow, or red-toned high-viscosity substance mainly used for building and repairing their hives. For thousands of years, propolis has been used in most civilized societies for various medicinal purposes [18,19]. The composition of industrial propolis from honey bees and stingless bees varies depending on the geographical locations in which bees and their vegetation are distributed; furthermore, its composition also varies depending on the climates and collection season of propolis [19]. Among the components of propolis, phenolic metabolites of plants are known to possess various biological activities including antioxidant activity [20]. Thus propolis rich in phenolic antioxidants has great potential to find utility in food, cosmetics, and medicines [20,21].

Korean propolis from various areas had high total phenolic content and strong antioxidant activity; the propolis from Cheongju had high contents of caffeic acid and caffeic acid phenethyl ester (CAPE) [22]. An ethanolic extract of Korean propolis provided ten phenylpropanoic acid esters, such as CAPE, caffeic acid benzyl ester, caffeic acid ethyl ester, ferulic acid benzyl ester, ferulic acid 3′,3′-dimethylallyl ester, 3,4-dimethoxycaffeic acid cinnamyl ester, *p-*coumaric acid cinnamyl ester, *p-*coumaric acid benzyl ester, cinnamic acid phenethyl ester, and cinnamic acid cinnamyl ester [23]. The components of Korean propolis, such as CAPE and quercetin, displayed potent antioxidant activities in vitro assays, and they inhibited tube formation and growth of human umbilical vein endothelial cells, supporting their potential anti-angiogenic activities [24]. Oral administration of Korean propolis attenuated oxidative stresses and neuronal degenerations induced by kainic acid in Sprague–Dawley rats, involving adenosine A1 receptor modulation [25].

The purpose of this study was to evaluate the antioxidant activity of Korean propolis in human epidermal keratinocytes exposed to airborne PM_10_. The ethanol extract of Korean propolis was divided into a hydrophilic fraction and a lipophilic fraction, and their effects on cell viability, ROS production, lipid peroxidation, and glutathione levels in human HaCaT keratinocytes were compared in the presence or absence of PM_10_. For the hydrophilic fraction, which was found to have relatively low toxicity and high antioxidant activity, component analysis and evaluation of the biological activity of the component were additionally performed. The results of this study suggested that Korean propolis, when properly purified, has the potential to be developed as a cosmetic material that helps to safely and effectively alleviate the skin toxicity of atmospheric particulate matter.

## 2. Materials and Methods

### 2.1. Reagents

Standardized fine dust (PM_10_-like, European standard ERM-CZ120), CAPE, caffeic acid, *p-*coumaric acid, and ferulic acid were purchased from Sigma-Aldrich (St. Louis, MO, USA).

### 2.2. Preparation of the Total Extract and Fractions of Propolis

Propolis was purchased in Andong, Gyeongsangbuk-do, Korea. Propolis raw material (60 g) was extracted with ethanol (600 mL) at room temperature for 4 days. After filtering, the filtrate was concentrated using a rotary evaporator under reduced pressure to obtain the total extract (23 g). The total extract was solvent-fractionated using equal volumes of water and methylene chloride and each fraction was evaporated under reduced pressure to obtain the hydrophilic fraction (0.6 g), the lipophilic fraction (18.5 g), and insoluble material (2.6 g).

### 2.3. High-Performance Liquid Chromatography with Photodiode Array Detection (HPLC-DAD)

HPLC-DAD analysis of the total extract of propolis and its fractions was performed using Waters Alliance HPLC system (Waters, Milford, MA, USA) consisting of e2695 separation module and 2996 photodiode array detector. A Hector-M C_18_ column (4.6 mm × 250 mm, 5 μm) (RS Tech Co., Daejeon, Korea) was used as the stationary phase. A mixture of 0.1% phosphoric acid (solvent A) and acetonitrile (solvent B) was used as the mobile phase with the changing composition: 0–30 min, a linear gradient from 0 to 100% B; 30–40 min, 100% B; 40–45 min, a linear gradient from 100 to 0% B. The flow rate of the mobile phase was set at 1.0 mL min^−1^, and the sample injection volume was 10 μL.

### 2.4. Cell Culture and Treatments

An immortalized human keratinocyte HaCaT cell line (CLS Cell Lines Service GmbH, Eppelheim, Germany) established by Dr. Norbert E. Fusenig [26] was cultured in a closed incubator at 37 °C in humidified air containing 5% CO_2_. The growth medium was DMEM/F-12 medium (GIBCO-BRL, Grand Island, NY, USA) supplemented with 10% fetal bovine serum, 100 U mL^−1^ penicillin, 100 µg mL^−1^ streptomycin, 0.25 µg mL^−1^ amphotericin B, and 10 μg mL^−1^ hydrocortisone. For each experiment, cells were seeded on 96-well, 12-well, or 6-well culture plates (SPL Life Sciences, Pocheon, Korea) and cultured for 24 h prior to various treatments. The total extract, its fractions, and individual compounds were treated alone or in combination with PM_10_ (200 μg mL^−1^) for 48 h to determine cell viability and lipid peroxidation, or for 60 min to measure ROS production.

### 2.5. Cell Viability Assay

HaCaT cells were seeded in 96-well culture plates at 4 × 10^3^ cells/well and maintained in a 200 μL culture medium for 24 h. After various treatments for 48 h, the cells were washed with phosphate-buffered saline (PBS) to remove residual extract, compound, and PM_10_, and their viability was measured using 3-(4,5-dimethylthiazol-2-yl)-2,5-diphenyl tetrazolium bromide (MTT) [27]. MTT (Sigma-Aldrich) was dissolved in PBS (5 mg mL^−1^) and diluted 5 times with a culture medium to the final concentration of 1 mg mL^−1^. The medium was dispensed by 100 μL per well in a 96-well plate and incubated at 37 °C for 2 h. After discarding the medium, cells were washed with PBS. The dye accumulated inside cells were extracted using 100 μL of dimethyl sulfoxide per well and the absorbance of the extracts was measured at 570 nm using a Spectrostar Nano microplate reader (BMG Labtech GmbH, Ortenberg, Germany).

### 2.6. Cellular ROS Production Assay

Cellular ROS production was assessed by using 2′,7′-dichlorodihydrofluorescein diacetate (DCFH-DA) [28]. The cells were plated onto 12-well culture plates at 1.4 × 10^5^ cells/well for 24 h. Cells were pre-labeled with 10 μM DCFH-DA (Sigma-Aldrich) for 30 min. After various treatments for 60 min, cells were washed twice with PBS and the fluorescence images of cells were obtained with a LEICA DMI3000 B microscope (Leica Microsystems GmbH, Wetzlar, Germany). For quantitative analysis, cells were lysed with 150 µL of the lysis buffer (1% sodium dodecyl sulfate (SDS), 20 mM Tris-Cl, 2.5 mM EDTA, pH 7.5), and the cell lysates were centrifuged with an Eppendorf centrifuge 5418R (Eppendorf, Barkhausenweg, Hamburg, Germany) at 14,500× *g* for 15 min to obtain the supernatant. The fluorescence intensity (excitation at 485 nm and emission at 538 nm) of the supernatants was measured with a Gemini EM fluorescence microplate reader (Molecular Devices, Sunnyvale, CA, USA).

Flow cytometry was additionally used to analyze intracellular ROS production. After various treatments, the adherent cells were detached from the culture plates using a trypsin-EDTA solution. Cells were centrifuged down with a Combi 408 centrifuge (Hanil, Daejeon, Korea at 316× *g* for 3 min, washed with PBS, and suspended in PBS. Flow cytometry for the cell suspension was conducted using BD FACSCalibur and data were analyzed using BD CellQuest (BD Biosciences, San Jose, CA, USA). Data are presented by the ratio (%) of cells with high DCFH-DA fluorescence due to intracellular ROS production to the total gated cells.

### 2.7. Lipid Peroxidation Assay

Cellular lipid peroxidation was assessed using 2-thiobarbituric acid (TBA) [29]. Cells were seeded at 2 × 10^5^ cells per well in a 6-well plate and cultured for 24 h. After various treatments with a test material in combination with PM_10_ (200 μg mL^−1^) for 48 h, cells were washed twice with PBS and lysed with 150 µL of the lysis buffer (1% SDS, 20 mM Tris-Cl, 2.5 mM EDTA, pH 7.5). The cell lysates were centrifuged with an Eppendorf centrifuge 5418R at 14,500× *g* for 15 min to remove cell debris and PM_10_. The mixture of 100 μL cell lysate (200 μg protein), 50 μL 1.0% *m-*phosphoric acid, and 350 μL 0.9% TBA (Sigma-Aldrich) was heated at 95 °C in a water bath for 45 min. The reaction was also run with 100 to 400 nM 1,1,3,3-tetramethoxypropane (Sigma-Aldrich) as a donor of malondialdehyde (MDA) to construct a standard curve. The limit of detection for the fluorometric assay has been determined to be 5 nM. After cooling to room temperature, 500 μL n-butyl alcohol was added to the mixture, vortex-mixed, and then the mixture was centrifuged to separate into two layers. The fluorescence intensity of the n-butyl alcohol layer (excitation at 544 nm and emission at 590 nm) was measured by using a Gemini EM fluorescence microplate reader. Data are presented as MDA levels corrected for protein contents.

In vitro experiments for lipid peroxidation were performed using the lysates of control HaCaT cells without any treatments. The HaCaT cell lysate (200 μg protein) was diluted with the lysis buffer and reacted with PM_10_ (200 μg mL^−1^) in the absence and presence of test material in a total volume of 200 μL in 1.5 mL Eppendorf tubes at 37 °C for 24 h. The reaction mixture was centrifuged to remove PM_10_. The supernatant (100 μL) was used in the assay of MDA levels as above.

### 2.8. Glutathione Assay

Glutathione contents were measured by a recycling assay [30]. After culturing and treatments in 6-well plates as above, cells were extracted using 5% meta-phosphoric acid (150 μL per well), followed by centrifuging with an Eppendorf centrifuge 5418R at 14,500× *g* for 15 min. The supernatant was used for the glutathione assay using a GSH/GSSG assay kit (product number GT40) from Oxford Biomedical Research (Oxford, UK). The total content of reduced glutathione (GSH) plus oxidized glutathione (GSSG) was measured using the extract as it is, and the GSSG content was quantified after pre-scavenging GSH in the extract with a pyridine derivative. Absorbance change due to reduction of 5,5′-dithio-bis-2-nitrobenzoic acid by GSH was measured at 412 nm, and a calibration curve prepared using a GSSG standard was used for the determination of glutathione contents. The GSH content was calculated by subtracting the GSSG content from the total content of GSH plus GSSG.

### 2.9. Assay for Free Radical Scavenging Activities

Spectroscopic methods were used to measure the scavenging activities of the sample against 2,2’-azinobis-(3-ethylbenzothiazoline-6-sulfonic acid) cation radical (ABTS^•^^+^) and 2,2-diphenyl-1-picrylhydrazyl radial (DPPH^•^) [31,32,33]. The ABTS^•^^+^ solution was prepared by mixing 0.54 mM ABTS solution (Sigma-Aldrich) and 0.27 mM potassium persulfate solution (Sigma-Aldrich) in equal volumes and allowing them to react for 24 h at room temperature (25 °C) in the dark. Each serial dilution of a plant-derived material or compound in ethanol (100 μL) was reacted with 0.27 mM ABTS^•^^+^ in water (100 μL) of at 25 °C for 3 min, followed by measurement of the absorbance at 734 nm with a BioRad Model 680 microplate reader (Bio-Rad Laboratories, Inc., Hercules, CA, USA). For the DPPH^•^ scavenging activity assay, a serially diluted sample in ethanol (100 μL) was mixed with 0.2 mM DPPH^•^ (Alfa Aesar, Ward Hill, MA, USA) in ethanol and reacted at 25 °C for 30 min. The absorbance was measured at 517 nm using a microplate reader.

### 2.10. Statistical Analysis

SigmaStat v.3.11 software (Systat Software Inc., San Jose, CA, USA) was used for the statistical analysis of the experimental data. Data are expressed as mean ± standard deviation (SD) of three or more independent experiments. The presence of significantly different group means among all groups was determined using a one-way analysis of variance (ANOVA) at the *p* < 0.05 level. Then, Duncan’s multiple range test was used to compare all groups to each other.

## 3. Results

### 3.1. Antioxidant Effects of Total Propolis Extract and Its Solvent Fractions in Cells

**Figure 1:**
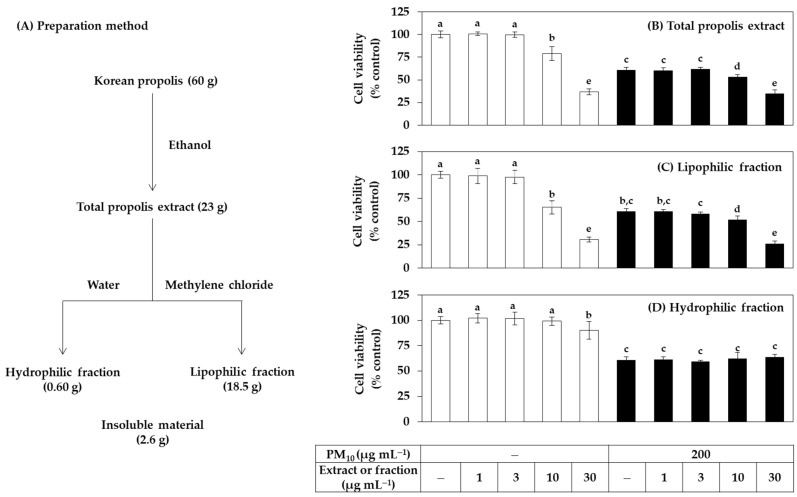
Effects of total propolis extract and its solvent fractions on the viability of human HaCaT keratinocytes exposed to PM_10_. In (**A**), the total ethanolic extract of Korean propolis was divided into a hydrophilic and a lipophilic fraction by solvent partition between water and methylene chloride. Cells were treated with the total extract (**B**), a lipophilic fraction (**C**), or a hydrophilic fraction (**D**) at the specified concentration alone or in combination with PM_10_ (200 μg mL^−1^) for 48 h. Cell viability was determined by the 3-(4,5-dimethylthiazol-2-yl)-2,5-diphenyl tetrazolium bromide (MTT) assay. Data are presented as mean ± SD (*n* = 5). (**D**) Duncan’s multiple range test was performed to compare all group means to each other. Groups that share the same letters (a–e) do not have significantly different means at the *p* < 0.05 level.

The total extract of Korean propolis and its hydrophilic and lipophilic fractions were prepared as depicted in Figure 1A. The yield of the total extract obtained by immersing the propolis raw material in ethanol was about 38.3%. The ratio of the hydrophilic fraction and the lipophilic fraction obtained by solvent fractionation of the total extract with water and methylene chloride was 1:31, and most of the extraction components were included in the lipophilic fraction.

In the first cell experiment, the effect of the total extract and its fractions on the viability of HaCaT cells in the presence or absence of PM_10_ exposure was investigated. The treatment concentration of PM_10_ was 200 μg mL^−1^, which was selected in the previous study [34]. Cells were treated with extracts or fractions alone or in combination with PM_10_, and cell viability was measured after 48 h. As shown in Figure 1B–D, the total propolis extract significantly reduced the cell viability at 10 μg mL^−1^ or higher, and the lipophilic fraction showed slightly stronger cytotoxicity than the total extract. However, the water fraction only reduced the cell viability by 10% at 30 μg mL^−1^, but not at the lower test concentrations. That is, the cytotoxicity of the hydrophilic fraction was relatively weak compared to the total extract and the fat-soluble fraction. As expected, PM_10_ exposure reduced cell viability by 40%, but neither the total extract nor the two fractions had any mitigating effect. The total extract and the lipophilic fraction further reduced the viability of PM_10_-exposed cells above 10 μg mL^−1^ because of their toxicity. On the other hand, the hydrophilic fraction did not change the viability of PM_10_-exposed cells due to its weak toxicity.

**Figure 2:**
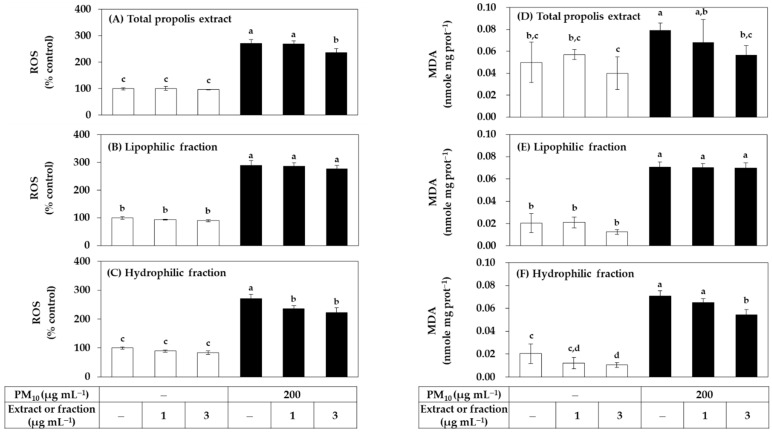
Effects of total propolis extract and its fractions on the reactive oxygen species (ROS) production and lipid peroxidation in HaCaT keratinocytes exposed to PM_10_. Cells were treated with the total extract (**A**,**D**), a lipophilic fraction (**B**,**E**), or a hydrophilic fraction (**C**,**F**) at the specified concentration alone or in combination with PM_10_ (200 μg mL^−1^) for 60 min for the determination of ROS production, or 48 h for the determination of lipid peroxidation. In (**A**–**C**), cells were pre-labeled with 10 μM 2’-7’dichlorofluorescin diacetate (DCFH-DA) for 30 min, and fluorescence of oxidized probe due to cellular ROS production was determined after treatments with the extracts and/or PM_10_. In (**D**–**F**), lipid peroxidation levels of cell lysates were determined by the thiobarbituric acid (TBA) assay. Data are presented as malondialdehyde (MDA) levels corrected for protein contents. Data are presented as mean ± SD (*n* = 4 for (**A**–**C**); *n* = 3 for (**D**–**F**)). Duncan’s multiple range test was performed to compare all group means to each other. Groups that share the same letters (a–d) do not have significantly different means at the *p* < 0.05 level.

Since both the total extract of propolis and its hydrophilic and lipophilic fractions had no cytotoxicity at a concentration of 3 μg mL^−1^ or less, it was evaluated whether they could reduce PM_10_-induced oxidative stress in cells in a low concentration range. HaCaT cells were treated with the total extract or fraction at a concentration of 1 μg mL^−1^ or 3 μg mL^−1^ alone or in combination with PM_10_. ROS production was measured after 60 min of PM_10_ exposure and lipid peroxidation was measured after 48 h. As shown in Figure 2, the total extract significantly reduced ROS production at 3 μg mL^−1^. Among the two fractions, the hydrophilic fraction significantly reduced ROS production at 1–3 μg mL^−1^, and the lipophilic fraction did not show such effects. The total extract and the hydrophilic fraction also significantly inhibited lipid peroxidation at 3 μg mL^−1^, and the lipophilic fraction had no such effects.

**Figure 3:**
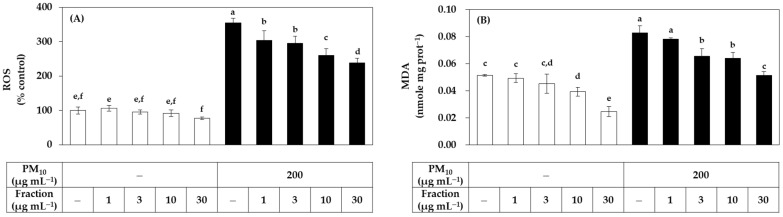
Effects of a hydrophilic fraction of propolis on the ROS production and lipid peroxidation in HaCaT keratinocytes exposed to PM_10_. Cells were treated with a hydrophilic fraction at the specified concentration alone or in combination with PM_10_ (200 μg mL^−1^) for 60 min for the determination of ROS production, or 48 h for the determination of lipid peroxidation. In (**A**), cells were pre-labeled with 10 μM (DCFH-DA) for 30 min and fluorescence of the oxidized probe due to cellular ROS production was determined after treatments with the extracts and/or PM_10_. In (**B**), lipid peroxidation levels of cell lysates were determined by TBA assay. Data are presented as MDA levels corrected for protein contents. Data are presented as mean ± SD (*n* = 4). Duncan’s multiple range test was performed to compare all group means to each other. Groups that share the same letters (a–f) do not have significantly different means at the *p* < 0.05 level.

Since the relatively low cytotoxicity of the hydrophilic fraction was seen in Figure 1, an additional experiment was conducted by extending the treatment concentration range of this fraction. As shown in Figure 3, the hydrophilic fraction inhibited PM_10_-induced ROS generation and lipid peroxidation in a concentration-dependent manner in the range of 1–30 μg mL^−1^. Combining the above results, it was suggested that the hydrophilic component of the propolis extract can relieve oxidative stress in cells exposed to PM_10_ more safely and effectively than the lipophilic component.

### 3.2. Analysis of Total Propolis Extract and Its Solvent Fractions

**Figure 4:**
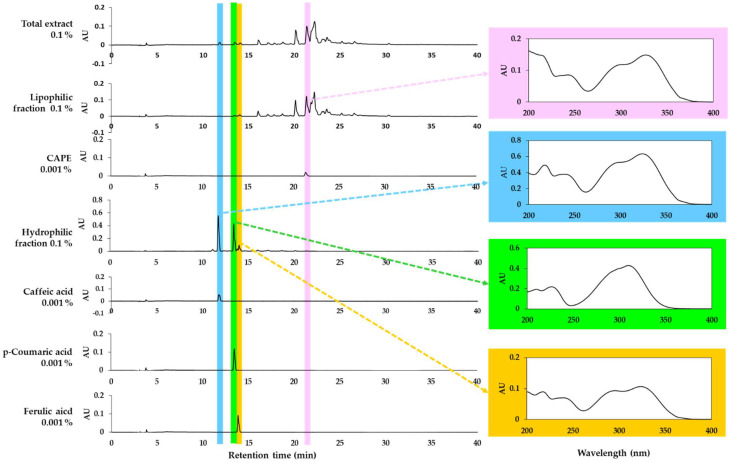
High-performance liquid chromatography-photodiode array detection (HPLC-DAD) analysis of the total extract of propolis and its solvent fractions. Authentic caffeic acid phenethyl ester (CAPE), caffeic acid, *p-*coumaric acid, and ferulic acid were used to identify the major peaks by comparing retention times and absorption spectra. Chromatograms detected at 310 nm and the absorption spectra of the designated peaks are shown.

HPLC-DAD analysis of the total extract of propolis and its lipophilic and hydrophilic fractions was performed. As shown in Figure 4, the total propolis extract and the two fractions show different phytochemical profiles. It was confirmed that one of the main components of the lipophilic fraction was CAPE. However, this study focused on the hydrophilic fraction based on the observed safety and effectiveness. The main peaks of the hydrophilic fraction were identified as caffeic acid, *p-*coumaric acid, and ferulic acid by comparing the retention times and absorption spectra of the standards. Among them, *p-*coumaric acid and ferulic acid are also partially included in the lipophilic fraction. The contents of caffeic acid, *p-*coumaric acid, and ferulic acid in the total extract were found to be similar to each other.

### 3.3. Antioxidant Effects of Phenylpropanoid Compounds of Propolis in Cells

**Figure 5:**
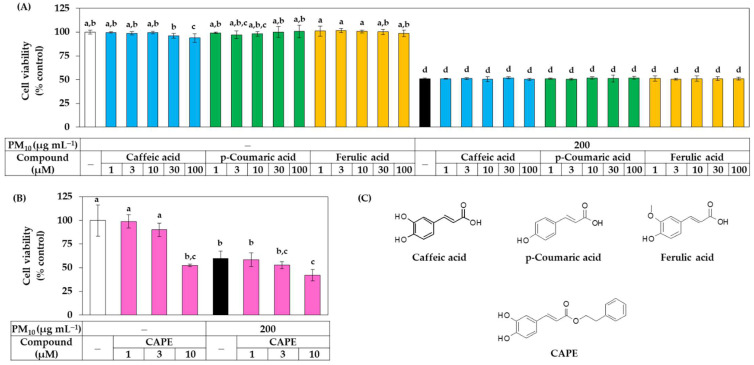
Effects of caffeic acid, *p-*coumaric acid, ferulic acid, and CAPE on viability in HaCaT keratinocytes exposed to PM_10_. In (**A**,**B**), cells were exposed to PM_10_ (200 μg mL^−1^) for 48 h in the absence and presence of each compound at the indicated concentrations. Cell viability was determined by the MTT assay. Data are presented as mean ± SD (*n* = 4 for (**A**); *n* = 5 for (**B**)). Duncan’s multiple range test was performed to compare all group means to each other. Groups that share the same letters (a–d) do not have significantly different means at the *p* < 0.05 level. In (**C**), the chemical structure of caffeic acid, *p-*coumaric acid, ferulic acid, and CAPE are shown.

Additional experiments were conducted to compare the biological activities of these three phenylpropanoid compounds. The effect of these compounds on the viability of HaCaT cells in the presence or absence of PM_10_ was investigated. As shown in Figure 5A, caffeic acid slightly reduced the cell viability at 100 μM, but all three phenylpropanoid compounds were found to be non-toxic at most concentrations tested. These three compounds did not affect the cell viability under PM_10_ exposure conditions. As shown in Figure 5B, CAPE exhibited cytotoxicity that reduced the cell viability by 50% at a concentration of 10 μM and aggravated the toxicity of PM_10_, so it was excluded from subsequent experiments. The chemical structures of caffeic acid, *p-*coumaric acid, ferulic acid, and CAPE are shown in Figure 5C.

**Figure 6:**
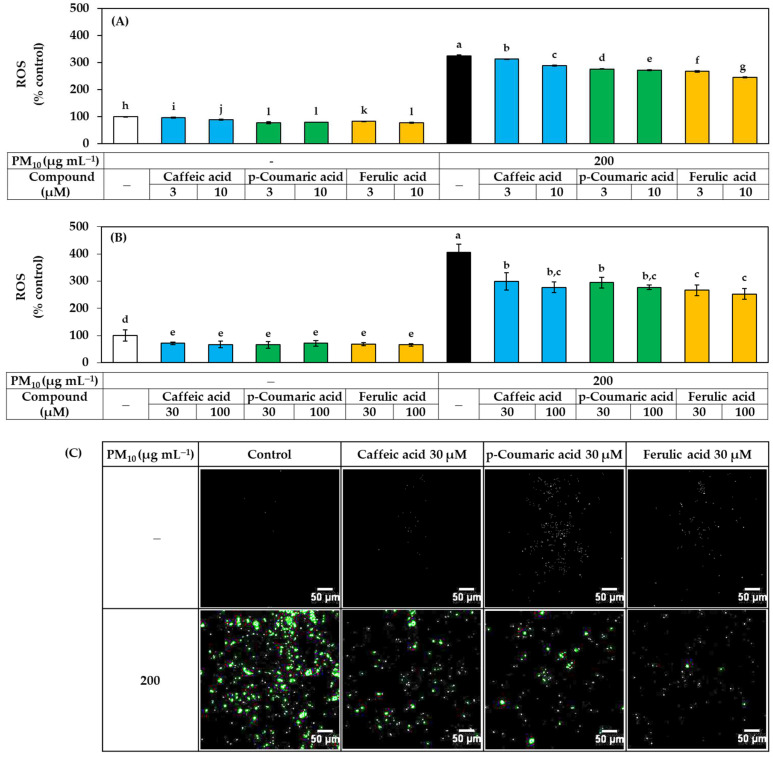
Effects of caffeic acid, *p-*coumaric acid, and ferulic acid on the ROS production in HaCaT keratinocytes exposed to PM_10_. Cells were labeled with DCFH-DA, treated with each compound at the indicated concentrations, and exposed to PM_10_ (200 μg mL^−1^) for 60 min or not. In (**A**,**B**), the fluorescence of the cell extracts was measured to quantitatively determine ROS levels. Data are presented as mean ± SD (*n* = 4). Duncan’s multiple range test was performed to compare all group means to each other. Groups that share the same letters (a–l) do not have significantly different means at the *p* < 0.05 level. Typical images of cells fluorescing due to ROS production are shown in (**C**).

The effects of these three compounds on ROS production and lipid peroxidation in HaCaT cells exposed to PM_10_ were further compared. As shown in Figure 6, each compound reduced ROS production in a concentration-dependent manner, and the effect was in the order of ferulic acid > *p-*coumaric acid > caffeic acid, especially in the low concentration range.

**Figure 7:**
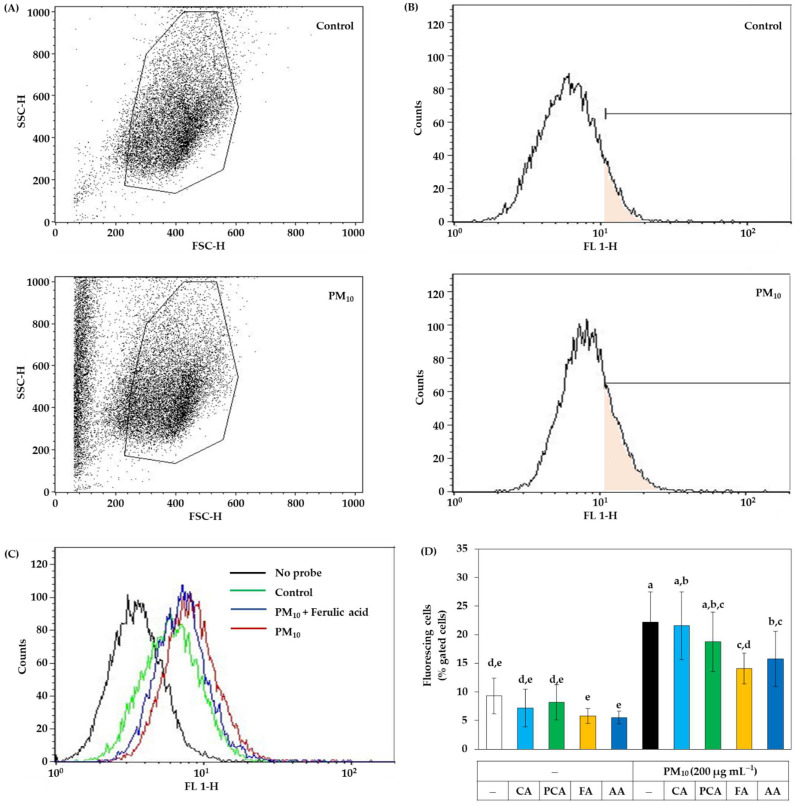
Flow cytometry for the ROS production in HaCaT keratinocytes exposed to PM_10_ in the absence and presence of caffeic acid (CA), *p-*coumaric acid (PCA), ferulic acid (FA), and ascorbic acid (AA). The adherent cells were labeled with DCFH-DA, treated with vehicle or each compound at 30 μM, and exposed to PM_10_ (200 μg mL^−1^) for 60 min or not. Cells were washed, detached, centrifuged down, and suspended in PBS for flow cytometry. (**A**) The gate was set to exclude the PM_10_ particles and cell aggregates. (**B**) The plots of the cell counts versus fluorescence intensity are shown with a mark to define fluorescing cells. (**C**) Typical effects of PM_10_ in the absence and presence of FA on the distribution of cells with different fluorescence levels. (**D**) The ratios (%) of fluorescing cells to the total gated cells are presented. Data represent mean ± SD (*n* = 3). Duncan’s multiple range test was performed to compare all group means to each other. Groups that share the same letters (a–e) do not have significantly different means at the *p* < 0.05 level.

Flow cytometry was additionally used to analyze intracellular ROS production without cell disruption. Despite washing cells with PBS, PM_10_ resides on cells and forms aggregates with cells. As shown in Figure 7A, PM_10_ treatment increased the counts of particles or cells with low forward scattering and high side scattering. Thus, the gate was set to exclude the particles and cell aggregates. Figure 7B shows the plots of the cell counts versus fluorescence intensity. Figure 7C shows typical plots for the cells with different treatments. In Figure 7D, the ratios (%) of cells with high fluorescence to the total gated cells were compared between cells treated with PM_10_ in the absence and presence of caffeic acid, *p-*coumaric acid, ferulic acids, and ascorbic acid (a positive control antioxidant) at 30 μM. The results indicated that PM_10_ increased ROS production in cells and the change was significantly inhibited by ferulic acid and ascorbic acid in the order. Caffeic acid and *p-*coumaric acid had no significant effects. Thus, the antioxidant effect of ferulic acid inhibiting intracellular ROS production was evaluated to be comparable to that of ascorbic acid by flow cytometry.

**Figure 8:**
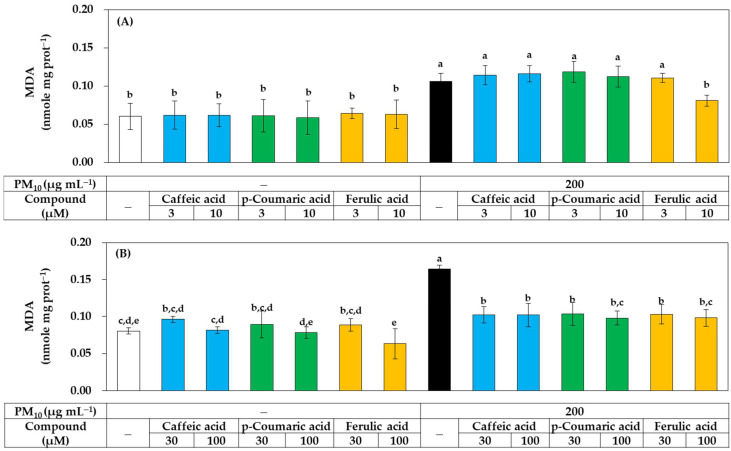
Effects of caffeic acid, *p-*coumaric acid, and ferulic acid on the lipid peroxidation in HaCaT keratinocytes exposed to PM_10_. Cells were treated with each compound at 3–10 μM (**A**) or 30–100 μM (**B**) alone or in combination with PM_10_ (200 μg mL^−1^) for 48 h. Lipid peroxidation levels of cell lysates were determined by TBA assay and data are presented as MDA levels corrected for protein contents. Data are presented as mean ± SD (*n* = 4). Duncan’s multiple range test was performed to compare all group means to each other. Groups that share the same letters (a–e) do not have significantly different means at the *p* < 0.05 level.

As shown in Figure 8, all three compounds at 30–100 μM significantly inhibited lipid peroxidation in PM_10_-exposed HaCaT cells, but at 10 μM, only ferulic acid showed a significant inhibitory effect, while the other two compounds had no significant effect. These results suggest that, although all three compounds have antioxidant activity that can relieve oxidative stress in cells, ferulic acid has relatively advantageous properties.

**Figure 9:**
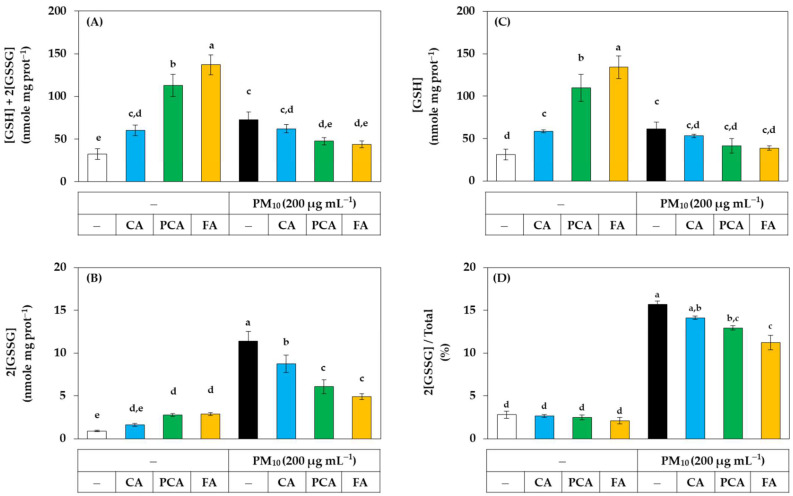
Effects of caffeic acid (CA), *p-*coumaric acid (PCA), and ferulic acid (FA) on the contents and ratios of glutathione (GSH) and glutathione disulfide (GSSG) in HaCaT keratinocytes exposed to PM_10_. Cells were treated with each compound at 30 μM and cultured in the absence or presence of PM_10_ (200 μg mL^−1^) for 24 h. The total contents of GSH plus GSSG (**A**) were subtracted by the GSSG contents (**B**) to calculate the GSH contents (**C**). The ratios of GSSG contents to the total contents were presented in (**D**). Data are presented as mean ± SD (*n* = 3). Duncan’s multiple range test was performed to compare all group means to each other. Groups that share the same letters (a–e) do not have significantly different means at the *p* < 0.05 level.

The effects of caffeic acid, *p-*coumaric acid, and ferulic acid on the redox balance of cells were examined by quantifying cell glutathione in the presence or absence of PM_10_. As shown in Figure 9A, in the absence of PM_10_, ferulic acid, *p-*coumaric acid, and caffeic acid increased the total glutathione content in the order. However, the increases in total glutathione caused by ferulic acid and *p-*coumaric acid were significantly inhibited by PM_10_. PM_10_ itself also significantly increased total glutathione, but this increase was inhibited by ferulic acid and *p-*coumaric acid. As shown in Figure 9B, in the absence of PM_10_, ferulic acid and *p-*coumaric acid slightly increased the content of oxidized glutathione (GSSG). More notably, PM_10_ increased GSSG content by more than 10-fold, and the increase was strongly inhibited by ferulic acid, *p-*coumaric acid, and caffeic acid in the order. As shown in Figure 9C, in the absence of PM_10_, ferulic acid, *p-*coumaric acid, and caffeic acid significantly increased the content of reduced glutathione (GSH) in this order. However, PM_10_ significantly inhibited the increase in GSH content by ferulic acid and *p-*coumaric acid. PM_10_ itself also slightly increased GSH content. As shown in Figure 9D, PM_10_ markedly increased the ratio of the GSSG content to the total glutathione content, and this change was significantly inhibited by ferulic acid and *p-*coumaric acid in the order. This complex phenomenon requires further study for interpretation but suggests that PM_10_ and phenylpropanoids, such as ferulic acid, may have diverse effects on the redox balance of cells. Nonetheless, these results demonstrate that the oxidative stress due to PM_10_ can be alleviated by phenylpropanoids, such as ferulic acid and *p-*coumaric acid.

### 3.4. Antioxidant Effects of Phenylpropanoid Compounds of Propolis In Vitro

**Figure 10:**
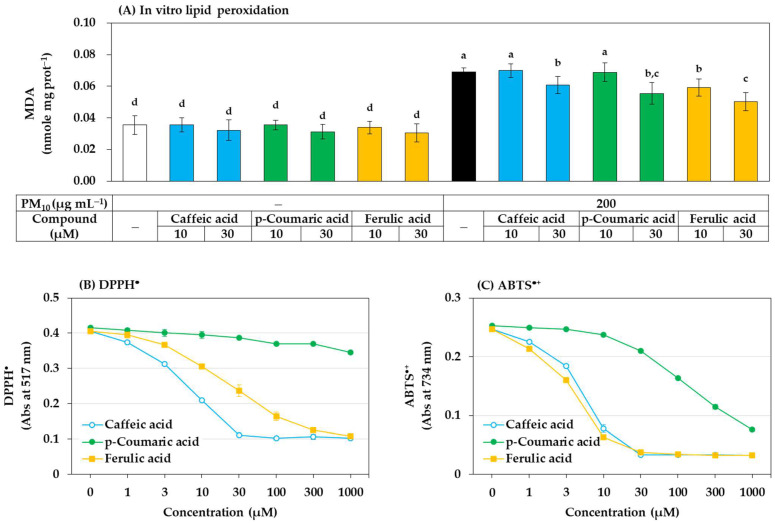
Effects of caffeic acid, *p-*coumaric acid, and ferulic acid on the lipid peroxidation of HaCaT cell lysate treated with PM10 in vitro, and their free radical scavenging activities against 2,2-diphenyl-1-picrylhydrazyl radical (DPPH^•^) and 2,2’-azinobis-(3-ethylbenzothiazoline-6-sulfonic acid) cation radical (ABTS^•^^+^) in vitro. (**A**) HaCaT cell lysate was treated with PM_10_ (200 μg mL^−1^) for 24 h in the absence or presence of a compound at the specified concentration. DPPH^•^ (**B**) and ABTS^•^^+^ (**C**) were reacted with each compound at different concentrations, and their remaining levels were measured by absorbance at 517 nm and 734 nm respectively. Data are presented as mean ± SD (*n* = 3). Duncan’s multiple range test was performed to compare all group means to each other. Groups that share the same letters (a–d) do not have significantly different means at the *p* < 0.05 level.

Among the above three phenylpropanoids, ferulic acid had the strongest antioxidant effect in preventing ROS generation, lipid peroxidation, and GSH oxidation in cells, followed by *p-*coumaric acid and caffeic acid. What is the mechanism? Possibly, these compounds might directly and chemically inhibit the oxidation reactions catalyzed by PM_10_. To examine this possibility, an in vitro experiment using the cell lysate was additionally performed. As shown in Figure 10A, when HaCaT cell lysate was exposed to PM_10_ in vitro, lipid peroxidation was induced, and strong inhibitory action was shown in the order of ferulic acid, *p-*coumaric acid, and caffeic acid. This trend matches the results obtained in cell experiments. Therefore, it is suggested that various phenylpropanoids, such as ferulic acid, can directly and chemically inhibit the oxidation reaction of cellular components by PM_10_.

In many studies, scavenging activity for free radicals, such as DPPH^•^ and ABTS^•^^+^ is measured to search for general antioxidants or to evaluate their antioxidant activity. The activities of caffeic acid, *p-*coumaric acid, and ferulic acid that scavenge two types of free radicals in vitro were compared. As shown in Figure 10B, among the three compounds, caffeic acid scavenged DPPH^•^ most strongly, followed by ferulic acid and *p-*coumaric acid. As shown in Figure 10C, the ABTS^•^^+^ scavenging activity of ferulic acid was slightly stronger than that of caffeic acid, and *p-*coumaric acid was much weaker than the other two compounds. No special correlation was found between their reactivity to DPPH^•^ or ABTS^•^^+^ and their inhibitory effect on the PM_10_-induced lipid peroxidation in vitro and in cells.

## 4. Discussion

This study showed the positive and negative effects of Korean propolis components on human epidermal keratinocytes exposed to PM_10_. The total extract of propolis and its lipophilic fraction were cytotoxic, which significantly reduced the viability of keratinocytes, whereas no such cytotoxicity was observed for its hydrophilic fraction. The hydrophilic fraction of the propolis extract showed antioxidant activity that inhibited cellular ROS production and lipid peroxidation induced by exposure to PM_10_, but the lipophilic fraction did not show such effects. Therefore, to use the propolis extract as a material for skin protection, it would be better to use it after removing harmful ingredients and enriching the active ingredients through a purification process rather than using it as it is.

Antioxidants that can directly or indirectly alleviate oxidative stress in cells are expected to be useful in reducing PM_10_-induced skin inflammation and premature aging [17]. They can inhibit the production of ROS, scavenge ROS already produced, or enhance cellular antioxidant capacity by stimulating the gene expression of antioxidant enzymes mediated by nuclear factor erythroid 2-related factor 2 [35]. We have shown that various phenolic compounds contained in terrestrial and marine plants, such as punicalagin, (−)-epigallocatechin-3-gallate, chlorogenic acid, and dieckol reduce ROS production, lipid peroxidation, and inflammatory responses in HaCaT cells exposed to PM_10_ [16,34,36]. In this study, it was additionally reported that caffeic acid, *p-*coumaric acid, and ferulic acid, as phenolic compounds contained in propolis, have antioxidant actions to reduce PM_10_-induced oxidative stress.

Plants are a source of various phytochemicals with diverse biological activities that are potentially useful to improve skin health and beauty [37,38,39,40]. Some phytochemicals act as either antioxidants or pro-oxidants and that show either cytotoxic or cytoprotective effects depending on their chemical nature and treatment conditions [41,42,43,44]. Therefore, it is important to select a phytochemical suitable for use and to optimize its biological activity by treating it at an optimal concentration for an optimal time. Since propolis contains various phenolic components derived from plants, various biological activities can be expected [18,19,20].

As observed in this study, the total extract of propolis has relatively strong cytotoxicity, and several previous studies reported the anti-proliferative and pro-apoptotic effects of propolis in various cancer cells. The extracts of the propolis from Chile, Brazil, Thailand, and Egypt have been shown to exert anti-proliferative and pro-apoptotic effects in various human cancer cell lines, such as mouth epidermoid carcinoma (KB), colon adenocarcinoma (Caco-2), androgen-insensitive prostate cancer (DU-145), laryngeal epidermoid carcinoma (Hep-2), cervical adenocarcinoma (HeLa), pulmonary adenocarcinoma (A549), and prostate cancer (PC3) cell lines [45,46,47,48]. A component of propolis, CAPE, was shown to induce apoptosis through activation of caspase-3, down-regulation of Bcl-2, and up-regulation of Bax in human leukemic HL-60 cells [49]. Caffeic acid and CAPE reduced glutathione levels and induced apoptosis of HeLa cells but not of Chinese hamster lung V79 fibroblast cells, suggesting that these compounds preferentially induce apoptosis of malignant cells through modulation of cellular redox state [50]. In the current study, CAPE showed strong toxicity to keratinocytes, and caffeic acid was also relatively more toxic than *p-*coumaric acid and ferulic acid, which matched well with previous studies.

On the other hand, the protective action of propolis extract in various cells has been reported. Uruguayan propolis induced the expression of endothelial nitric oxide synthase (NOS) while inhibiting endothelial NADPH oxidase, and thus it was suggested that the propolis can provide a cardiovascular protective benefit by increasing nitric oxide (NO) bioavailability in the endothelium [51]. Water extract of Brazilian green propolis and its constituents, 3,4-di-*O*-caffeoylquinic acid, 3,5-di-*O*-caffeoylquinic acid, chlorogenic acid, and *p-*coumaric acid exerted protective effects against the oxidative damage induced by glutathione depletion using L-buthionine-(S,R)-sulfoximine in cultured retinal ganglion cells, supporting its potential neuroprotective effects [52]. Ethyl acetate extracts of propolis from Algerian regions effectively scavenged free radicals, prevented lipid peroxidation, and inhibited myeloperoxidase activity, whereas its petroleum ether and chloroform extracts inhibited anticholinesterase activity [53,54]. Italian propolis with high polyphenolic components effectively inhibited lipid peroxidation of linoleic acid in SDS micelles and showed appropriate ultraviolet (UV) absorptivity to be used as broad-spectrum UVB and UVA photoprotection sunscreens [55]. However, there have been few studies focusing on the effect of propolis extracts on oxidative stress induced by atmospheric pollution.

In the current study, the hydrophilic fraction of the propolis extract was shown to have relatively weak cytotoxicity than the lipophilic fraction and have antioxidant activity to inhibit ROS generation and lipid peroxidation, suggesting that the hydrophilic fraction is more useful for protecting the skin from air pollution. This study also showed that the cytotoxicity of caffeic acid, *p-*coumaric acid, and ferulic acid contained in the hydrophilic fraction was very low compared to CAPE, which is one of the main components of the lipophilic fraction, and that these three compounds can mitigate the oxidative stress induced by PM_10_ in keratinocytes. The total content of caffeic acid, *p-*coumaric acid, and ferulic acid in the hydrophilic fraction of the propolis extract was estimated to be 14.2% (caffeic acid, 10.2%; *p-*coumaric acid, 3.63%; ferulic acid, 0.38%) by HPLC-DAD analysis, and 30 μg mL^−1^ of this fraction corresponds to 4.3 μg mL^−1^ (24.2 μM) of the compounds; caffeic acid, 3.1 μg mL^−1^ (17.0 μM); *p-*coumaric acid, 1.1 μg mL^−1^ (6.6 μM); ferulic acid, 0.1 μg mL^−1^ (0.6 μM). The results of this study suggest that these three compounds in combination are partially responsible for the antioxidant activity of the hydrophilic fraction. 

Despite the structural similarity of caffeic acid, *p-*coumaric acid, and ferulic acid, their cytotoxicity, reactivity to different ROS, antioxidant activity, and other biological activities are very different [56,57]. In our current study, the DPPH^•^ scavenging activity was caffeic acid > ferulic acid > *p-*coumaric acid, ABTS^•^^+^ scavenging activity was ferulic acid > caffeic acid > *p-*coumaric acid, and inhibitory activity against PM_10_-induced lipid peroxidation was ferulic acid > *p-*coumaric acid > caffeic acid. Maurya et al. reported that ferulic acid showed weaker DPPH^•^ scavenging activity and stronger ABTS^•^^+^ scavenging activity than caffeic acid, and that ferulic acid inhibited 2,2′-azobis (2-methylpropionamidine) dihydrochloride (AAPH)-induced lipid peroxidation more effectively [58]. The results of both studies agree well with each other. Although caffeic acid exhibits stronger ROS scavenging activity than many other phenylpropanoids [56], it can act as a pro-oxidant rather than as an antioxidant under certain conditions [58]. It is presumed that ferulic acid has a higher probability to act as an efficient antioxidant rather than as a pro-oxidant in general cellular contexts, compared to caffeic acid and *p-*coumaric acid.

There are several methods that can measure PM_10_-stimulated ROS in cellular models, but each has its own advantages and disadvantages. Direct measurement of the fluorescence of adherent cells can minimize changes that may occur during the extraction process, but unwashed black PM_10_ may affect the fluorescence measurement. In contrast, the method of extracting fluorescent probes from cells can more effectively remove PM_10_ and aggregated cell debris by centrifugation, but cannot completely rule out changes in the extraction process. Therefore, we used the two methods to complement each other. In flow cytometry, PM_10_ can be mistaken for small cells or can form cell aggregates, which can alter light scattering by cells. Gate settings that exclude cells highly affected by PM_10_ may distort the cell population to be analyzed, reducing the reliability of experimental data. There is also a high risk of PM_10_-generated cell aggregates blocking the flow cell and causing mechanical failure. Thus, a special caution is required when using flow cytometry for the analysis of PM_10_-treated cells.

## 5. Conclusions

This study investigated the effects of extracts of Korean propolis, its hydrophilic and lipophilic fractions, and several major components on the viability and oxidative stress of keratinocytes exposed to PM_10_. In particular, the hydrophilic fraction and phenylpropanoid compounds, such as ferulic acid, contained in this fraction showed antioxidant action to inhibit PM_10_-induced ROS generation, lipid peroxidation, and glutathione oxidation, suggesting their potential to be used as cosmetic and dermatological active ingredients. Since propolis contains both cytoprotective and cytotoxic components, a purification process to improve its safety and efficacy is required for use in skin protection. Additional in vivo experiments and clinical studies are needed to apply the results of this study to cosmetic or dermatology.

## Data Availability

The data presented in this study are available in the article.
